# Community Indicators: Assessing the Impact of Alcohol Use on Communities

**DOI:** 10.35946/arcr.v35.2.04

**Published:** 2014

**Authors:** Andrea Flynn, Samantha Wells

**Affiliations:** **Andrea Flynn, Ph.D.**, *is a project scientist at the Centre for Addiction and Mental Health, Social and Epidemiological Research Department, London, Ontario, Canada.*; **Samantha Wells, Ph.D.**, *is a scientist at the Centre for Addiction and Mental Health, Social and Epidemiological Research Department, London, Ontario, Canada; adjunct assistant professor at the Western University, Department of Epidemiology and Biostatistics, London, Ontario, Canada; and assistant professor at the University of Toronto, Dalla Lana School of Public Health, Toronto, Ontario, Canada.*

**Keywords:** Alcohol use, abuse, and dependence, alcohol burden, problematic alcohol use, harmful drinking, alcohol-related harm, alcohol use patterns, alcohol effects and consequences, alcohol availability, risk factors, environmental impact, crime, community indicators, community monitoring, community epidemiology, data collection, public policy on alcohol

## Abstract

Community indicators are used to assess the impact of alcohol on communities. This article reviews the main data sources for community indicators, discusses their strengths and limitations, and discusses indicators used in reference to four main topics relating to alcohol use and problems at the community level: alcohol use, patterns, and problems; alcohol availability; alcohol-related health outcomes/trauma; and alcohol-related crime and enforcement. It also reviews the challenges associated with collecting community indicator data, along with important innovations in the field that have contributed to better knowledge of how to collect and analyze community-level data on the impact of alcohol.

In the United States and other countries around the world, researchers have long been interested in community-level measurement of population health in the form of community indicators. Community indicators are measures that communicate information about a given dimension of a community’s well-being (Besleme and Mullin 1997). In the United States, the current popularity of community indicators can be traced back to the social-indicators movement of the 1960s and 1970s (see Gross and Straussman 1974; Land and Spilerman 1975; MacRae 1985), which saw growing research attention paid to the measurement of social problems and issues such as divorce, crime, education, and social mobility. Although the social-indicators movement initially focused on issues at the national level, recognition of considerable regional and local variation in the prevalence and causes of social problems led to increased interest in measurement at the local level and, as such, the development of “community indicators.”

Community indicators that assess alcohol use and related harm are of great interest to community stakeholders and researchers. Alcohol use has been identified as a major risk factor for acute and chronic health harms and imparts economic, health, and social costs to individuals, communities, and societies (Rehm et al. 2009). Alcohol intoxication is linked to injury, violence, and traffic crashes (Edwards et al. 1994) and chronic alcohol use increases the risk of liver damage and various cancers, among other health harms (Edwards et al. 1994; Rehm et al. 2003; Room et al. 2005). National surveys have revealed a great deal of variability across different communities in the extent of alcohol use and related harms (Gruenewald et al. 1997). Thus, it may not be practical or fiscally responsible to base local prevention and intervention initiatives on national data that do not reflect patterns or problems within a particular community. Moreover, prevention, treatment, and enforcement activities are commonly enacted at the local level (Gruenewald et al. 1997). Therefore, community-level data on the impact of alcohol use that take into consideration the local economic, social, and policy context are key to guiding local decisionmaking and maximizing the effectiveness of prevention and intervention approaches.

Community indicators have been used extensively for a variety of purposes by both researchers and community stakeholders. For communities, indicator data can be used to inform priority-setting agendas by identifying specific concerns within a community, guide policy and education initiatives, monitor community status on a particular measure over time or in comparison with other communities, and evaluate programs or policies (Besleme and Mullin 1997; Gabriel 1997; Gruenewald et al. 1997; Mansfield and Wilson 2008; Metzler et al. 2008). Local-level data also are critical for justifying requests for funding and provide a powerful tool for resource allocation within communities (Mansfield and Wilson 2008). For researchers, community indicators are central for improving knowledge of factors influencing community well-being, advancing innovative theoretical models and analytical approaches for use in research and prevention planning (for example, see Holder 1998*a*), and monitoring and evaluating community prevention/intervention initiatives (Metzler et al. 2008).

This article provides an overview of community indicators of alcohol use and related harms, outlining common sources of community indicator data and highlighting the various challenges of collecting data on alcohol at the community level. The literature on community indicators of alcohol use and harms is expansive, spanning a large number of disciplines and extending back for numerous decades. As such, it is beyond the scope of this article to provide a comprehensive review of all the literature and measures pertaining to community indicators on alcohol. Rather, this article provides background information relevant to the use of community indicators in general and in relation to alcohol use and harms, providing examples of some of the most common measures used by alcohol researchers. In addition, the article mentions notable methodological and technological advances that have characterized this field of study over the past few decades, while highlighting the ongoing challenges faced by researchers and community stakeholders interested in assessing alcohol use and alcohol-related harm at the local level. This article draws on extensive knowledge regarding community indicator data on alcohol use and harms that has emerged from key community-based intervention trials, such as the Saving Lives project led by Hingson (Hingson et al. 1996), the Community Trials project led by Holder (Grube 1997; Holder 2000; Holder and Reynolds 1997; Holder and Treno 1997; Holder et al. 1997*a*, 1997*b*, 2000; Millar and Gruenewald 1997; Reynolds et al. 1997; Saltz and Stanghetta 1997; Treno and Holder 1997; Voas 1997; Voas et al. 1997), and the Communities Mobilizing for Change on Alcohol (CMCA) project led by Wagenaar (Wagenaar et al. 1994, 1999, 2000*a*, 2000*b*). The sections that follow outline some of the main community indicators emerging from this literature and other relevant research in reference to four main topics—alcohol use, patterns, and problems; alcohol availability; alcohol-related health outcomes/trauma; and alcohol-related crime and enforcement.

## What Is A Community?

A number of different definitions of community have been proposed and used in the social sciences since the 1800s (for a helpful overview of the various ways in which community has been defined historically, see Holder 1992). Generally speaking, the concept of community implies both geographic and social proximity. Gruenewald and colleagues (1997) define a community as “a contiguous geopolitical area over-seen by a common political structure with common policing and enforcement agencies and common educational and utility systems, and in which individuals are in daily physical contact for the purposes of economic and social exchange” (pp. 10–11). Holder (1992, 1998*b*) provides a similar definition based on a community-systems perspective and theoretically geared toward the prevention of alcohol problems. Community, in this context, is conceptualized as a dynamic, complex, and adaptive system consisting of “a set or sets of persons engaged in shared socio-cultural-politico-economic processes” (Holder 1998*b*, p. 12). This definition informs the theoretical premise that reducing alcohol use and alcohol-related problems requires a focus on the community system and structural factors influencing alcohol use rather than on individual-level treatment and prevention (Holder 1998*b*; Holder et al. 2005; Treno and Lee 2002).

Putting these definitions of community into practice when attempting to define and use community indicators is not without its challenges and has direct implications for data collection. When defining the boundaries of the community for the purpose of generating community indicators, it is necessary to consider data availability, methodological requirements of research (i.e., having sufficient cases for meaningful analyses), the catchment area in terms of service provision, other geographic boundaries according to which data are routinely collected by a community, and local stake-holder perspectives on their understanding of community (Gruenewald et al. 1997). These considerations do not always coincide (e.g., available data may not match the catchment area of interest to community stakeholders), making it necessary to weigh the relative importance of these factors when defining the boundaries of the community under study (Gruenewald et al. 1997).

## Data Sources for Community Indicators on Alcohol

Community indicators relating to alcohol use and harms are typically gleaned from two main types of data sources: (1) archival sources collected for purposes other than addressing research questions on the impact of alcohol on communities (e.g., data from police and hospital records; crash data from traffic safety databases); and (2) primary data collected by researchers for the purpose of assessing, understanding, and addressing alcohol use and related harms. These different sources of data have inherent advantages and disadvantages in terms of their utility for assessing the community-level impact of alcohol use.

### Archival Data

Archival data are an important source of community indicator data. Examples of these archival data sources include administrative and surveillance databases maintained by local city departments, community organizations, municipal/national agencies, schools, hospitals, and police/law enforcement departments, in addition to larger health data–recording systems and traffic crash databases (e.g., the Healthcare Cost and Utilization Project [HCUP] databases and the Fatality Analysis Reporting System [FARS]). A wide range of indicators produced from archival data are used to assess various alcohol-related issues and harms at the community level (examples and discussion of common indicators are presented in the section Community Indicators on Alcohol and Alcohol-Related Harm; see also the table).

A main benefit of using archival sources to produce community indicators is that they can be a cost-effective means of documenting alcohol use and harms, offering a large volume of retrospective data. In addition, unlike many of the constructs and measures used in social and epidemiological research, archival data often result in indicators that are straightforward, understandable, and of interest to the community, making them easier to use in community planning (Gabriel 1997; Gruenewald et al. 1997; Mansfield and Wilson 2008). Despite these advantages, there also are several limitations associated with using archival data to assess alcohol use/harms in a community. By definition, these data are not gathered for research purposes and thus raise concerns relating to both reliability and validity. Most notably, archival data are subject to various sources of measurement error consequent to the fact that they are not collected according to the systematic and rigorous procedures that characterize social and epidemiological research. In addition, for some measures, the involvement of alcohol may not be explicitly identified. For instance, hospital staff and police typically do not systematically record data on alcohol consumption as part of routine practice (Brinkman et al. 2001; Gruenewald et al. 1997; Stockwell et al. 2000). When alcohol data are recorded in community settings, they may be collected in an inconsistent manner, influenced by subjective judgments and local practices (Brinkman et al. 2001). These limitations affect the extent to which researchers can confidently use existing data such as hospital records or police data to assess alcohol involvement in injury or crime. Moreover, access to such data requires cooperation of local community agencies and/or municipal or regional departments, which may not be always possible.

Another important caveat relates to the use of archival data for conducting community comparisons. Differences across communities in policies and data recording systems (Gruenewald et al. 1997; Brinkman et al. 2001; Stockwell et al. 2000) can make it difficult to conduct comparisons across communities. For example, when using arrest data on alcohol-related crime such as public intoxication or disorderly conduct, the indicator will reflect the definition used by the police department (itself dependent on local or regional statutes) as well as on local enforcement capacity and practices, including levels of police discretion. Thus, data on arrests may not be directly comparable across communities, even if the communities themselves are well matched on demographic or other important baseline measures (Gruenewald et al. 1997). Changes in recording systems or policies also present problems for researchers interested in examining patterns over time within communities. For example, variation over time in the number of alcohol-related arrests may reflect changes in enforcement, recording practices, or policies rather than true variations in alcohol-related crime (Gruenewald et al. 1997).

Events with low levels of incidence present another challenge relating to use of archival data for assessing the impact of alcohol on communities. For instance, although alcohol-related morbidity and mortality are of great interest to communities, these types of indicators may be difficult to provide at the community level, particularly for smaller communities, because of their relatively low baseline rate. Moreover, in the case of health-related indicators, the problem of low incidence is compounded by the fact that most health-related harms associated with alcohol use are only partially attributable to alcohol (Rehm et al. 2003). Although researchers have developed approaches for estimating the proportion of a given outcome that is attributable to alcohol as a specific risk factor (i.e., the attributable fraction, AF) (see English et al. 1995; Martin et al. 2010; Rehm et al. 2003; Single et al. 1999; Stockwell et al. 2000; World Health Organization [WHO] 2000), these types of analyses require a large volume of data and are typically only conducted at higher levels of aggregation (e.g., State, Federal).

### Primary Data

Given that archival data often are unavailable or insufficient to assess alcohol use and harm at the community level, primary data are collected to enhance knowledge of the community-level impact of alcohol use (Gruenewald et al. 1997; Stockwell et al. 2000). Population or subpopulation surveys are the predominant source of primary data used to produce alcohol-related community indicators. Surveys offer the advantage of allowing researchers to define the constructs of interest and use psychometrically sound measures, including measures that have been used in other community-level, State, or Federal surveys, thereby facilitating comparisons. Surveys also permit the collection of self-report data that cannot be gleaned from archival data, such as individual-level alcohol use patterns; underage access to alcohol; and beliefs, attitudes, and perceptions surrounding alcohol. These data allow for individual and group-level risk factors to be determined and permit analyses on subpopulations of interest, such as adolescents or young adults (Gruenewald et al. 1997; Stockwell et al. 2000).

In some instances, it may be possible to extract community-level data from surveys conducted at higher levels of aggregation (e.g., State or national surveys). However, the time frames of State and national surveys often do not meet community or research needs. For example, timing of data collection is an essential factor when monitoring the impact of local policy changes or community initiatives, which may not coincide with national survey data collection (Mansfield and Wilson 2008). Moreover, when attempting to glean information from national or State-level surveys, sample sizes for smaller communities often are insufficient to permit valid conclusions about specific communities or population subgroups within a community (Gruenewald et al. 1997; Mansfield and Wilson 2008; Stockwell et al. 2000). For these reasons, surveys implemented at the community level are key to developing local indicators of alcohol use and harms. Surveys have been widely used in community-based research projects, including both general population surveys and surveys of particular population groups, such as college students (discussed below in Community Indicators on Alcohol and Alcohol-Related Harm; see also the table).

When conducting surveys to produce community indicators, it is necessary to consider the limitations of the survey method. Recent evidence suggests that population surveys can underestimate the prevalence of alcohol use and associated harms because of selection bias, response bias, and coverage bias (e.g., exclusion of homeless people) (Shield and Rehm 2012; see also Curtin et al. 2005; Dillman et al. 2002; Kempf and Remington 2007). The growth in use of voicemail, caller ID, cell phones, and do-not-call lists, along with a growing aversion to aggressive telemarketing (Galesic et al. 2006), have contributed to a notable decline in telephone survey response rates (Dillman et al. 2002; Hartge 1999; Kempf and Remington 2007; see also Galea and Tracy 2007). Young people may be particularly underrepresented in population surveys, given their high reliance on cell phones and nonuse of landlines (Blumberg et al. 2007). Large-scale surveys can also be expensive and time consuming to implement.

When collecting primary data on alcohol use and harms, it is also important to consider the limitations of self-report data on drinking behavior and harms associated with drinking. Although self-report data on alcohol use generally are believed to be adequately valid and reliable and are widely used in social and epidemiological research, they have been found to be susceptible to recall error as well as intentional distortion related in part to social desirability (Del Boca and Darkes 2003).

Despite these limitations, surveys are key to answering specific questions about alcohol use and harms in the absence of suitable archival data and are central for cross-validating data gleaned from other sources. Moreover, extensive work on conducting surveys as part of community prevention trials has led to important methodological and statistical innovations, producing advanced knowledge of how to design and analyze surveys better (see Murray 1998; Murray and Short 1995, 1996; Murray et al. 2004).

In addition to surveys, other forms of primary data used to produce community indicators include pseudo-patron studies designed to assess sales of alcohol to individuals appearing underage in both off-premise and on-premise alcohol outlets (see, for example, Freisthler et al. 2003; Saltz and Stanghetta 1997; Toomey et al. 2008; Treno et al. 2006; Wagenaar et al. 2000*a*) and roadside breath testing to assess drinking and driving (e.g., McCartt et al. 2009; Roeper and Voas 1998). These methods and their strengths and limitations are discussed in later sections on alcohol availability and crime/enforcement, respectively.

Overall, although primary data, particularly surveys, allow for the use of psychometrically sound measures, they suffer from potential biases that researchers must take into account when assessing the impact of alcohol use on a community. Alternatively, archival data sources can provide useful data on alcohol’s effects on local communities but require careful interpretation and application and do not always allow researchers to answer questions of interest. Each data source thus offers unique strengths and limitations, such that triangulation of both types of data is a common approach taken by alcohol researchers when assessing the impact of alcohol on communities.

## Community Indicators on Alcohol and Alcohol-Related Harm

Table 1 provides a summary of common community indicators of alcohol use and related harms measured in community-based research. These indicators are organized into four broad areas: alcohol use, patterns, and problems; alcohol availability; alcohol-related health outcomes/trauma; and alcohol-related crime/enforcement. Although this table does not provide an exhaustive list of all possible measures used to assess alcohol use and alcohol-related harm at the community level, it provides common measures used in community research (see Saltz et al. 1992). For each category, examples of indicators produced using archival and primary data sources are provided, and general strengths and limitations associated with these data are noted.

### Alcohol Use, Patterns, and Problems

At the community level, indicators of alcohol use, patterns, and problems commonly are produced from individual-level self-report (i.e., survey) data. Existing community-based studies have examined a wide range of self-report measures of alcohol use, including, for example, lifetime drinking, drinking frequency, heavy episodic drinking (or binge drinking) and hazardous or harmful drinking, alcohol problems, and alcohol dependence (see Dent et al. 2005; Flewelling et al. 2005; Harrison et al. 2000; Hawkins et al. 2009; Perry et al. 1996, 2000, 2002; Saltz et al. 2009, 2010; Spera et al. 2010; Wagenaar et al. 2006; see table 1). It is beyond the scope of this article to discuss the many different instruments used and all of the methodological challenges associated with measuring self-reported drinking and problems. Choice in how to measure indicators of use, patterns, and problems will depend on the research question being asked and the population under examination. The strengths and limitations of various specific measures of alcohol consumption have been discussed extensively in the literature (see Dawson 2003; Gmel et al. 2006*a*; Graham et al. 2004; Greenfield 2000; Rehm 1998; Rehm et al. 1999), and recommendations for measurement have been put forward elsewhere (see Dawson and Room 2000).

Drinking behavior among youth often is of particular interest to both researchers and communities. Evidence suggests that youth are more likely than adults to engage in risky patterns of drinking (Adlaf et al. 2005) and to experience harms from drinking, including harms to brain development, physical health, financial well-being, and social life (Adlaf et al. 2005; Kolbe et al. 1993; Toumbourou et al. 2007; White and Swartzwelder 2004). Moreover, drinking at a young age can become an ingrained pattern of behavior, with youth who engage in risky drinking being more likely to exhibit problem drinking later in life (Jefferis et al. 2005). For these reasons, measuring alcohol use and alcohol-related problems among youth often is prioritized in prevention and early-intervention initiatives designed to reduce harm from alcohol at both the individual and community levels (see DeJong et al. 2009; Nelson et al. 2010). The well-known prevention initiative CMCA (Wagenaar et al. 1994, 1999, 2000*a*, *b*) is notable for its focus on community-level strategies for reducing alcohol use and problems among youth and its development of indicators of alcohol use and harms to evaluate program effectiveness.

Surveys on youth drinking have commonly captured these populations in their educational environments, including elementary, high school, and college or university settings. The priority of addressing alcohol use among college students is well evidenced by the NIAAA’s Rapid Response to College Drinking Problems initiative, which produced recommendations for reducing heavy drinking by this subgroup (see DeJong et al. 2009; Nelson et al. 2010). Alcohol use, patterns, and problems have been measured in the implementation and evaluation of alcohol prevention trials in school and college settings (see reviews by Saltz 2011 for college-based prevention approaches and Stigler et al. 2011 for elementary and high school programs). Examples of measures of alcohol use and problems among college and school-age students include self-reported alcohol use (i.e., measures of frequency of drinking, drinking patterns, and binge drinking) (Flewelling et al. 2005; Harrison 2000; Hawkins et al. 2009; Perry et al. 1996, 2000, 2002; Saltz et al. 2009, 2010), the incidence and likelihood of intoxication at off-campus drinking establishments (Saltz et al. 2010), age of onset of drinking (Hawkins et al. 2009), and perceptions and experiences of negative consequences associated with drinking (Flewelling et al. 2005; Saltz et al. 2009, 2010). Significantly, although surveys of college and university students may provide communities with estimates of alcohol use, patterns, and problems among this segment of the population, these surveys are inherently limited to the sampling frame of youth attending these institutions. As a result, they fail to capture youth from the broader community not attending educational institutions and thus cannot offer community prevalence data for that age range.

With respect to archival data on alcohol use, this type of information is less commonly available at the community level compared with higher levels of aggregation. Most notable in this regard is the use of sales data to examine per capita alcohol consumption. WHO (2000) has recommended that alcohol use among populations be monitored using reliable estimates of per capita alcohol consumption derived from alcohol sales data, in addition to monitoring through population surveys of alcohol use. Sales data commonly have been used at the State, regional, and Federal levels to examine the link between per capita alcohol consumption and various health harms, including suicide (Kerr et al. 2011*b*, Landberg 2009), mortality and morbidity (Kerr et al. 2011*a*; Nordstrom and Ramstedt 2005; Polednak 2012), and traffic crashes (Gruenewald and Ponicki 1995). These types of analyses, however, generally are restricted to large populations (Dawson 2003) and thus are less applicable to alcohol researchers interested in community indicators (i.e., measures below the State level of aggregation), in part as a result of the low base rate of harms at the community level and in part from challenges associated with obtaining sales data at the community level compared with the State level.

### Availability

Measuring the availability of alcohol at the community level is essential for assessing the impact of policies designed to reduce alcohol use and alcohol-related harms (see Babor et al. 2003). Availability commonly is measured in terms of commercial access (including alcohol outlet density, days and hours of sales, and price of alcohol) as well as social access (i.e., informal sources of alcohol, such as peers).

With respect to commercial access, although the evidence on the effects of limiting alcohol outlet density on alcohol consumption is somewhat mixed (see Livingston et al. 2007), studies generally have found significant positive relationships between alcohol outlet density and a range of problems at the community level, including rates of violence, drinking and driving, motor vehicle accidents, medical harms, and crime (Britt et al. 2005; Campbell et al. 2009; Gruenewald and Remer 2006; Gruenewald et al. 2006; Livingston et al. 2007; Toomey et al. 2012). Evidence also suggests a positive relationship between days (Middleton et al. 2010) and hours (Hahn et al. 2010) of sale and alcohol consumption and alcohol-related harms (see also Edwards et al. 1994). Alcohol prices and taxes are inversely related to alcohol consumption and heavy drinking (Chaloupka et al. 2002; Edwards et al. 1994; Osterberg 2004; Wagenaar et al. 2009), although the extent of the impact of price changes depends to some extent on cultural context (i.e., drinking norms) and prevailing social and economic circumstances, among other factors (Osterberg 2004; see also Babor et al. 2003). Researchers have used indicators of commercial access to evaluate whether changes in State policies have an impact on alcohol use/problems in communities (see Babor et al. 2003; Edwards et al. 1994; Hahn et al. 2010; Middleton et al. 2010).

Community indicators of economic availability commonly are produced using archival data sources, including alcohol price and tax (excise and sales) data from State departments and alcohol-control boards, although the quality of these data and their utility for research at the community level varies substantially across States (Gruenewald et al. 1997). Archival data on retail alcohol prices are difficult to obtain at the State level, and even more so at the community level. Evidence suggests that available data are prone to substantial measurement error (Young and Bielinska-Kwapisz 2003), leading many researchers to rely on tax data instead. When making comparisons across communities or over time, researchers generally also prefer to use tax rates over price data to avoid conflating price differences with differing tax rates across space and over time. Liquor licensing information from alcohol-control boards commonly is used to generate indicators of commercial availability—namely, number of outlets/population rates and concentration of on- and off-premise outlets (Sherman et al. 1996; see also Gruenewald et al. 1997). However, counts of active licenses represent only an indirect measure of alcohol availability and can underestimate alcohol sales (Gruenewald et al. 1992). Geographic Information System (GIS) mapping has emerged as an innovative means of generating community indicators of outlet density (including off- and on-premise outlets) and to examine alcohol outlet density and locations in relation to alcohol-related problems, such as assaults and sale of alcohol to minors (see Gruenewald et al. 2002; Millar and Gruenewald et al. 1997).

One major caveat relating to measures of commercial access to alcohol is that archival data obscure who is making purchases, who is consuming the alcohol purchased, and how (in what patterns) the alcohol is being consumed. Therefore, important information about risky drinking behavior (i.e., binge drinking) and populations who engage in such behavior remains unknown from data on alcohol availability. This limitation is particularly salient for measuring drinking among youth, who commonly obtain alcohol from social rather than commercial sources (see Wagenaar et al. 1993).

In light of this limitation, and the fact that early prevention of alcohol use and alcohol-related problems is often a high priority for communities and researchers, other data collection strategies have been implemented to measure access to alcohol among youth. Access surveys involving pseudo-underage youth purchase attempts have produced indicators of youth commercial access, often as part of the evaluation of community prevention initiatives (see Chen et al. 2010; Grube 1997; McCartt et al. 2009; Paschall et al. 2007; Perry et al. 1996, 2000, 2002; Toomey et al. 2008; Wagenaar et al. 1994, 1999, 2000*a*, *b*). Self-reported social access to alcohol has also been measured in school or community surveys of youth, with participants asked to report on sources from which they obtain alcohol (i.e., commercial [on- or off-premise outlets] versus social [friends, family, etc.] sources) (see Dent et al. 2005; Harrison et al. 2000; Hearst et al. 2007; Jones-Webb et al. 1997; Wagenaar et al. 1994). Some studies also have examined perceived availability of alcohol among youth (Flewelling et al. 2005; Perry et al. 1996, 2000, 2002; Treno et al. 2008).

### Health Outcomes/Trauma

As stated previously, evidence reveals a strong and consistent association between alcohol consumption and a variety of negative health outcomes, including morbidity, early mortality, and increased risk of trauma such as burns, falls, drowning, and injury from interpersonal violence (Cherpitel 1995; Gmel et al. 2006*b*; Rehm et al. 2003, 2006; Treno et al. 1997). Collectively, alcohol-related health harms and traumas impose notable demands on local emergency and hospital services. Documenting alcohol-related morbidity, mortality, and trauma is thus often a priority for communities and researchers, with such research informing initiatives geared toward preventing alcohol-related harm and efforts to reduce health costs.

Both archival and primary data have been used to produce community indicators relating to fatal and nonfatal alcohol-involved health harms. Data sources and types of indicators emerging from these data include (1) hospital data, used to produce indicators of hospitalizations and emergency department (ED) visits associated with acute or chronic alcohol use; (2) traffic fatality data, used to estimate alcohol involvement in crashes; and (3) household or subpopulation surveys, used to generate indicators from self-reported data on alcohol-involved injuries (including violence). As shown in table 1, each of these data sources has strengths and limitations pertaining to their utility for producing community indicators on alcohol-related harms.

#### Hospital and ED Data

Archival hospital data allow for documentation of cases of alcohol-related health outcomes and trauma requiring urgent or emergent care. Such data can provide powerful information for use by communities (e.g., in educational or prevention campaigns) because of their severity and corresponding psychological impact (Stockwell et al. 2000). Despite this appeal, notable challenges exist to using archival data to produce community indicators on health outcomes and trauma associated with alcohol. First, as stated above, one of the major caveats with measuring alcohol-related mortality and morbidity at the community level is the rarity of cases (Giesbrecht et al. 1989; Stockwell et al. 2000), meaning that there may be insufficient numbers for meaningful analysis at the community level. Second, it often is quite difficult to obtain access to hospital or ED data within communities, particularly data of reasonable quality for developing valid and reliable estimates. Third, it often is challenging or impossible to determine the extent of alcohol involvement in health outcomes. As previously noted, many chronic health harms associated with alcohol, including those leading to hospitalization and mortality, are only partially attributable to this risk factor (Rehm et al. 2003). In terms of emergency cases, archival data frequently do not capture alcohol involvement (Giesbrecht et al. 1989; Stockwell et al. 2000). Blood alcohol concentration (BAC) is not routinely assessed in hospitals or urgent-care centers in relation to traumatic presentations, given that staff generally are operating under time and resource constraints that preclude systematic testing for alcohol use. Staff also may be hesitant to make conclusions about intoxication because of insurance and liability concerns (Giesbrecht et al. 1989, 1997; Stockwell et al. 2000; Treno and Holder 1997). As a result, archival data of emergency cases likely underestimate the role of alcohol in trauma requiring emergent care. In cases where BAC is recorded, determining the role of alcohol in a traumatic event is complicated by time elapsed since the incident and by alcohol consumed after the incident (Young et al. 2004).

In the face of challenges associated with lack of documentation of alcohol involvement in archival data, researchers commonly turn to surrogate measures of alcohol-related trauma. Such measures have been well studied using international data. For instance, Young and colleagues (2004) found that being male, unmarried, younger than age 45, and presenting at EDs in the late night or early morning hours on Fridays, Saturdays, or Sundays were most highly associated with alcohol consumption prior to injury (based on BAC and self-reported alcohol consumption within 6 hours prior to injury). The strongest predictor of alcohol-related injury was time of day of presentation (odds ratio of 4.92 for presentations occurring between midnight and 4:59 a.m.). It follows that, in the absence of reliable BAC data, proxy measures that take into account time-of-day presentation and demographic variables may offer a means for estimating alcohol-related trauma in a community (Brinkman et al. 2001; Treno et al. 1996). Such estimates require access to medical records that include time-of-day presentation and detailed demographic information.

Archival data on hospitalizations and ED visits are becoming more readily available for use in the development of community indicators. For example, the Healthcare Cost and Utilization Project (see Steiner et al. 2002, http://www.hcup-us.ahrq.gov/) consists of a series of health care databases that provide data on inpatient, ambulatory, and ED cases for community hospitals in participating States since 1988. These databases permit research on topics such as diagnoses; procedures; mortality; cost of health services; access to health care programs; and treatment outcomes at the national, State, and local levels (http://www.hcup-us.ahrq.gov/). Some participating States allow the release of hospital and patient-level geographic data that may permit analysis at the community level (Steiner et al. 2002).

Researchers have also produced indicators on alcohol-involved trauma at the community level from ED surveys, involving the collection of interview and breathalyzer data from ED patients (see Cherpitel 1994 and 1993 for reviews of ED studies; see also Busset al. 1995; Cherpitel et al. 2009; Holder et al. 2000; Treno and Holder 1997). Cherpitel (1995) measured alcohol-related problems and injuries or illnesses for which emergency medical care was sought in a countywide representative study of ED data. When comparing these data to a general population sample, Cherpitel (1995) found no difference in frequency of drunkenness related to injury between the two samples, suggesting that ED surveys may be a useful approach for measuring these issues. However, obtaining ED cooperation and producing representative ED samples is a notable challenge faced by researchers when endeavoring to conduct ED surveys (Holder et al. 2000).

#### Traffic Fatality Data

Alcohol-related traffic fatalities are an important form of trauma in the community-indicator literature on alcohol-related harm. Consistent evidence confirms that alcohol is a leading cause of traffic crashes, particularly those resulting in fatal and nonfatal injuries (Hingson and Winter 2003). Research has demonstrated that the relative risk of fatal injury and fatal crash involvement rises with increasing driver BAC (see the classic Grand Rapids study by Borkenstein et al. [1974] and subsequent studies by Hurst [1973]; Krüger and Vollrath [2004]; Mathijssen and Houwing [2005]; Mayhew et al. [1986]; McCarroll and Haddon [1962]; Perrine et al. [1971]; Zador [1991]; and Zador et al. [2000]). Relative risk data such as these have been widely used to support alcohol safety legislation, including the lowering of BAC driving limits (see review by Mann et al. 2001).

The FARS (formerly the Fatal Accident Reporting System) (see http://www.nhtsa.gov/FARS), initially established in 1975, is a reliable database of all fatal crashes in the United States and includes the BACs of drivers involved in fatal crashes. When chemical tests of driver BACs are not performed in fatal crashes, FARS provides imputed data (see Subramanian 2002). FARS data can be disaggregated to the level of the county (see Voas et al. 1998; Williams 2006). Studies using FARS or State traffic safety department databases have generated indicators of various levels of driver BAC associated with traffic fatalities (e.g., Hingson et al. 2005, et al. 2006; Wagenaar and Wolfson 1995). However, fatal crashes are relatively rare events (Voas et al. 1997), and thus aggregation of events over a long time period may be needed to produce sufficient cases for analysis at the community level (e.g., see Wagenaar et al. 2000*a*).

Researchers commonly also use fatal single-vehicle nighttime crashes as a surrogate for alcohol-involved traffic fatalities, which can be a useful strategy when data on alcohol involvement in crashes are unavailable for the community of interest or too few cases have been documented. These data have been shown to be a reliable proxy for alcohol-related fatalities. They often are available from local or State sources (e.g., police departments or departments of transportation) and, depending on the size of the community, may occur in sufficient numbers for analysis (see Hingson et al. 1996; Roeper and Voas 1998; Treno et al. 2006; Wagenaar and Holder 1991; Wagenaar et al. 2000*a*, 2006). Nevertheless, caution is warranted when interpreting traffic crash data, particularly in the absence of BAC data, given the myriad of other factors that stand to be involved in crashes, including road conditions, speeding, and use of seat belts. The use of multiple data sources for triangulation of data (Gruenewald et al. 1997) can help overcome the limitations of any one measure of alcohol-involved vehicle crashes.

#### Population Survey Data

Population or community surveys are used to measure self-reported alcohol-related health outcomes and trauma. An advantage of these surveys is that they can detect events not resulting in fatalities or hospital admissions (Gruenewald et al. 1997). These data are thus useful for documenting less severe cases, which are more common than fatal or near-fatal cases. However, the number of self-reported events (e.g., injury) may still be insufficient for analysis, particularly in small communities. General limitations of population surveys apply to these data, including the cost and time required to conduct them, as well as reporting and coverage biases that may result in underestimates of alcohol-related harms.

#### Crime/Enforcement

Both primary and archival data sources have been used to generate measures of alcohol-related crime in communities. At the community level, household, telephone, and school surveys have been conducted to measure various self-reported crimes, including driving under the influence (DUI) (e.g., Clapp et al. 2005; Saltz et al. 2009; Wagenaar et al. 2006), underage alcohol purchases (e.g., Harrison et al. 2000), alcohol-related violence (Greenfield and Weisner 1995), and public drunkenness (Greenfield and Weisner 1995). The general strengths and limitations of surveys and self-report measures of alcohol use have been discussed previously. Therefore, this section will focus on roadside surveys and arrest data.

Roadside surveys involve stopping motorists at roadside checkpoints for the purpose of collecting breath alcohol measurements. Two key purposes of roadside surveys are to track drinking and driving trends and to evaluate alcohol safety programs (Lange et al. 1999; Lestina et al. 1999). The majority of roadside studies conducted to track trends in drinking and driving have occurred at the national level (e.g., in the United States, Canada, Britain, Germany, Sweden, Norway, Belgium, and the Netherlands) (see Lacey et al. 2008; Lestina et al. 1999; Lund and Wolfe 1991; Voas et al. 1998; Wolfe 1974 for information on the U.S. National Roadside Surveys). These national surveys typically do not provide sufficient data at the community level for assessment of local drinking and driving because of the exclusion of smaller communities and/or roadways with low daily traffic counts (Voas et al. 1998). At the community level, roadside surveys primarily have been used in the evaluation of community prevention trials (e.g., McCartt et al. 2009; Roeper and Voas 1998). They allow researchers to assess changes in drinking-and-driving behavior in relation to prevention campaigns when fatality and crash data are unavailable (Roeper and Voas 1998). In instances where fatality and crash data are available, roadside survey data may still be useful to confirm that changes in crash data reflect valid changes in drinking-and-driving behavior rather than other changes not related to alcohol consumption (e.g., roadway improvements) (Roeper and Voas 1998).

Two main strategies are used to implement roadside surveys at the community level: (1) “piggybacking” on existing police sobriety check points; and (2) using roadside check points dedicated entirely to research. In both instances, cooperation of local police is imperative, which may create a challenge in communities lacking widespread support for the research (Howard and Barofsky 1992). In addition to the notable cost associated with conducting roadside surveys, there are several limitations and challenges associated with this method of data collection (Lestina et al. 1999). For example, many high-BAC drivers are able to avoid roadside survey check points by driving alternate routes, resulting in underestimates of local levels of drinking and driving (Lestina et al. 1999). Drivers also may refuse to provide a breath sample, and these people may be likely to have higher BACs than those who consent to a breath test (Lestina et al. 1999). Conversely, overestimates of impaired driving may occur if roadways characterized by high volumes of alcohol-related crashes are targeted for surveys (Lestina et al. 1999). In evaluations of alcohol-safety programs (and other alcohol interventions), it is necessary to compare the intervention community with a comparison community in which the program was not implemented to determine whether changes in drinking and driving can be attributed to the intervention. However, finding adequate comparison sites can be a challenge, given the need for a community with similar population characteristics and policies and the fact that comparison (“non-experimental”) communities may have their own campaigns to reduce drinking and driving (see Voas 1997).

Arrest data on DUI as well as other alcohol-related offenses also represent valuable indicators for communities. Numerous researchers have used archival police and justice records to produce community indicators of alcohol-related crimes, including DUI, liquor law violations, assault, public drunkenness, and disorderly conduct (e.g., Breen et al. 2011; Duncan et al. 2002; Sherman et al. 1996; Treno et al. 2006; Wagenaar et al. 2000*a*) (see table 1). When using archival data to assess levels of alcohol-related crime, it is important to recognize that such arrests represent only offenses brought to the attention of the police that they have acted upon. Some criminal events (e.g., violent crime) are not commonly reported to the police, or there may be insufficient cause for police to file an arrest report (Brinkman et al. 2001). Moreover, by definition, arrest data are dependent on local and State statutes and also are highly sensitive to enforcement capacity and practices as well as operational changes and recording practices, including police discretion (Gruenewald et al. 1997). These factors are thus critical to consider when making comparisons over time or across communities. As noted previously, changes in alcohol-related arrests can represent changes in actual crime, changes in enforcement or recording practices, or changes in policies and laws (Gruenewald et al. 1997). In some instances, confounding variables (such as police discretion in making arrests) are difficult if not impossible to measure.

Another problem with police data is that for many types of crime (e.g., violence), police do not formally measure alcohol involvement (i.e., through a breath test). Although some research has measured alcohol-involved crime through archival records of cases that police have flagged for alcohol involvement (Wagenaar et al. 2000*a*), these data are unlikely to be systematic and rely in large part on police discretion (see discussion by Brinkman et al. 2001). To partially address such concerns, surrogate measures have been used to produce indicators of alcohol-related crime from archival data. For example, nighttime assaults have been used as a proxy for alcohol-related violence, given that temporal data are likely to be recorded in police records and violent assaults during nighttime hours have a high likelihood of being alcohol related (Brinkman et al. 2001).

Indicators of enforcement are also related to measurement of alcohol-related crime at the community level. Some investigators have measured enforcement activities in community-based research projects, often for the purpose of evaluating policy changes or prevention efforts (e.g., Grube 1997; McCartt et al. 2009; Voas, Holder and Gruenewald 1997; see also Wagenaar and Wolfson 1995) (see table 1). Indicators of enforcement can provide communities with data on enforcement capacity and, if tracked over time, can allow for an assessment of the impact of enforcement on reducing alcohol-related crime.

## Conclusion

Measuring alcohol use and harm in communities is complex and requires researchers to make choices and find creative ways of assessing the local-level impact of alcohol. The data source and indicator used will depend on data availability, the purpose of the research (e.g., to provide a community with descriptive data versus evaluation of an intervention), and, in many cases, community support for the research to facilitate access to archival data or cooperation in primary data collection efforts.

Whether using archival or primary data to produce community indicators, it is important for both researchers and community stakeholders to be aware of the strengths and potential limitations of the data. They must also recognize the value of combining data from multiple sources when making conclusions about the impact of alcohol on communities. Indeed, many community-based projects have relied on both primary and archival data to assess alcohol use and harms in communities and to evaluate the impact of intervention initiatives. Triangulation of indicators is key for validating measures and thus drawing accurate conclusions about research findings.

Despite the limitations and challenges associated with assessing alcohol use and alcohol-related harms at the community level, many significant advances have been made in the field, including important advances in statistical methods (e.g., Murray 1998; Murray and Short 1995, 1996; Murray et al. 2004), refinement of surrogate measures (e.g., Treno et al. 1994, 1996, 1997), and spatial analysis (e.g., Gruenewald et al. 2002; Millar and Gruenewald 1997). Another example of an innovative approach that currently is being employed to develop community indicators involves use of a mobile research laboratory to collect social, epidemiological, and biological data in diverse communities in the province of Ontario, Canada. Led by a multidisciplinary team of researchers, this project involves collection of local data and the development of a community indicator database relating to mental health and addictions in participating communities, including indicators of alcohol use and harms (see Wells et al. 2011).

Building on these types of innovations and the rich history of social indicators in the United States, a number of communities recently have sought to develop comprehensive community indicator systems consisting of data on a range of factors (e.g., social, economic, and environmental) to allow a detailed examination of influences on community well-being (Besleme and Mullin 1997; Ramos and Jones 2005). National initiatives such as the 2008 Community Health Status Indicators (CHSI) project (see Heitgerd et al. 2008; Metzler et al. 2008; see also www.communityhealth.hhs.gov), the Community Assessment Initiative (http://www.cdc.gov/ai/index.html), and the National Neighborhood Indicators Partnership (http://www.neighborhoodindicators.org), for example, have sought to improve access to local data and inform use of data in planning efforts and evaluation of health policies and interventions. At the international level, the Community Indicators Consortium, established in 2003, represents one of the most extensive efforts to engage stakeholders from around the world and to document and share knowledge on community indicators (see Ramos and Jones 2005; http://www.communityindicators.net). Some projects included in the Community Indicators Consortium database of indicator projects specifically include risky alcohol consumption as part of their examination of community well-being (see http://www.communityindicators.net). These types of initiatives suggest that community indicators, including indicators of alcohol use and harm, will continue to grow in the coming years as an area of interest and innovation.

Community indicators are certainly not a panacea for either investigators or community stakeholders. However, when produced with a thorough understanding of the local community system and through thoughtful application of advanced methodological knowledge, they can serve as a powerful tool for understanding, assessing, and addressing alcohol-related problems within their local context.

## Figures and Tables

**Table:** Examples, Strengths and Limitations of Community Indicators from Archival and Primary Data Sources

**Indicator Category**	**Indicators from Archival Sources**			**Indicators from Primary Data Sources**		
	**Examples of Indicators**	**Strengths**	**Limitations**	**Examples of Indicators**	**Strengths**	**Limitations**
**Alcohol use, patterns and problems**	Per capita alcohol consumption	Generated from available sales data	Does not capture patterns of access or use Excludes “surrogate” alcohols (homemade, illegal, alcohol not intended for consumption) Data may not be available at the local level (depends on catchment area of research and definition of “community”)	Self-reported drinking behavior and problems (youth, adults) - age at first use - drinking prevalence - drinking volume - heavy episodic drinking (i.e., binge drinking) - hazardous or harmful drinking Alcohol dependence	Offer individual- and group-level data unavailable from archival sources that can be aggregated to community level, including drinking pattern Ability to implement scientifically valid and reliable measures employed in other communities and other levels of aggregation (State, Federal) for comparison purposes	General limitations of surveys and self-report measures - high cost of surveys - possible biases (selection bias, social desirability bias, recall bias, coverage bias)
**Alcohol availability**	Formal access - number of active outlet licenses per 100,000 population - concentration/spatial distribution of outlets - excise taxes on alcoholic beverages - price of alcoholic beverages	Data on outlet licenses are generally maintained with good geographic specificity by Alcohol Control Boards	Data do not capture sales to minors	Alcohol purchase attempts at alcohol outlets by pseudo-underage customers	Capture events not visible in archival data and not affected by self-report biases	Persuasiveness of results potentially undermined by the fact that buyers are actually of legal age
			Data do not capture social access			
					Useful in evaluations of strategies to reduce youth access to alcohol	
			Data do not capture differences between outlets with respect to sales (e.g., small outlets versus large outlets)			
			Community estimates may be affected by migratory patterns and purchases in communities of non-residence			
			Price data difficult to obtain			
	Social access			Self-report data collected from underage youth on ability to purchase alcohol at alcohol outlets Self-report data from underage youth on social sources of alcohol (friends, family members, bought by someone else, took from someone else’s home)	Provides data unavailable from archival sources Data on a high-risk group unavailable from archival data sources	General limitations of self-report data General limitations of self-report data and surveys Additional concerns with coverage bias for telephone surveys due to high rates of cell phone use among youth and young adults
**Alcohol-related health and trauma**	Hospital discharge data - rates of direct alcohol mortality or morbidity: alcohol cardiomyopathy, alcohol cirrhosis of liver, alcoholic psychoses, accidental ethyl alcohol poisoning, etc. - rates of indirectly-related alcohol deaths: certain malignant tumors, cirrhosis, pancreatitis, etc. Nighttime presentations of trauma from violence or traffic accidents (surrogate measures) Alcohol-involved traffic crashes Single-vehicle nighttime traffic crashes	Capture serious health/trauma outcomes – strong impact for communities Nighttime emergency department (ED) presentations and nighttime single vehicle traffic crashes are reliable surrogates of alcohol-involved trauma	Low base rates of mortality from alcohol at the community level Multiple causes of death often poorly recorded in archival data Proportion of mortality/morbidity events attributable to alcohol difficult to estimate at the community level Hospital/ED cases capture only the most severe cases Blood alcohol concentrations (BAC) not routinely recorded in hospital/emergency settings BAC not always measured in injury-producing/fatal crashes Fatal crashes rare at community level	Self-reported health harms and trauma experiences related to alcohol ED surveys - BAC measurement - self reported alcohol consumption prior to ED presentation	General strengths of surveys and self-report data BAC data provides objective measurement of alcohol involvement in injury presentations to ED Self-reported alcohol consumption shown to be valid measure of alcohol use	General limitations of self-report data and surveys Difficulty obtaining permission for ED surveys
**Alcohol-related crime**	Calls to police for nighttime assaults Calls to emergency medical services for alcohol-related injury Calls to police for public drunkenness or disorderly contact Arrest rates for driving under the influence Arrest rates for nighttime assaults Alcohol-related arrests as a percentage of total arrests	If cooperation can be obtained, arrest or EMS records are a cost-effective source of data that is meaningful to community members	Heavily dependent on police enforcement and accuracy in recording Difficult to determine if changes are due to changes in police enforcement, valid changes in crime, or prevention programs In community prevention trials or when communities are interested in comparisons, different statutes or operational policies affect ability to compare communities Arrests represent only a proportion of offenses – underestimates harm	Self-reported crime - alcohol consumption prior to driving/driving while intoxicated - violence perpetration after drinking Roadside survey data - BAC readings	Self-reported crime captures incidents not reported to police BAC provides an objective measure of alcohol consumption	General limitations of self-report data Challenges of implementing roadside surveys - can be difficult to obtain police cooperation - high cost - generally not random (not representative of community) - can be difficult to find appropriate comparison communities
